# Quality of life at the end of life

**DOI:** 10.1186/1477-7525-5-51

**Published:** 2007-08-03

**Authors:** Paula Diehr, William E Lafferty, Donald L Patrick, Lois Downey, Sean M Devlin, Leanna J Standish

**Affiliations:** 1 Department of Biostatistics, University of Washington, Seattle, Washington, 98195, USA; 2 Department Health Services, University of Washington, Seattle, Washington, 98195, USA; 3 Bastyr University, Kenmore, Washington, 98028, USA

## Abstract

**Background:**

Little is known about self-perceived quality of life (QOL) near the end of life, because such information is difficult to collect and to interpret. Here, we describe QOL in the weeks near death and determine correlates of QOL over time, with emphasis on accounting for death and missing data.

**Methods:**

Data on QOL were collected approximately every week in an ongoing randomized trial involving persons at the end of life. We used these data to describe QOL in the 52 weeks after enrollment in the trial (prospective analysis, N = 115), and also in the 10 weeks just prior to death (retrospective analysis, N = 83). The analysis consisted of graphs and regressions that accounted explicitly for death and imputed missing data.

**Results:**

QOL was better than expected until the final 3 weeks of life, when a terminal drop was observed. Gender, race, education, cancer, and baseline health status were not significantly related to the number of “weeks of good-quality life” (WQL) during the study period. Persons younger than 60 had significantly higher WQL than older persons in the prospective analysis, but significantly lower WQL in the retrospective analysis. The retrospective results were somewhat sensitive to the imputation model.

**Conclusion:**

In this exploratory study, QOL was better than expected in persons at the end of life, but special interventions may be needed for persons approaching a premature death, and also for the last 3 weeks of life. Our descriptions of the trajectory of QOL at the end of life may help other investigators to plan and analyze future studies of QOL. Methodology for dealing with death and the high amount of missing data in longitudinal studies at the end of life needs further investigation.

## 1.0 Background

Quality of life (QOL) can be viewed as people's “perceptions of their position in life in the context of their particular culture and value systems, and in relation to their personal goals, expectations, standards and concerns.” QOL can be known only to the individual concerned, and reflects an evaluation of circumstances both intrinsic and extrinsic. [1-3] It is important to understand and improve both the quality of care and the quality of life at the end of life. [4,5] Trends in QOL at the end of life are poorly understood because it is difficult to obtain such information on a timely basis. It is also difficult to interpret the QOL findings that are available, because of death and missing data. [6,7] In this paper we used preliminary data from an ongoing randomized trial of complementary and alternative medicine (CAM) at the end of life. Patients received up to two CAM visits each week, and the research staff collected QOL data after every two visits. This paper's goal is to characterize QOL over time in the persons enrolled to date, and to determine correlates of QOL over time. We emphasize methods of accounting for death and missing values in these longitudinal data.

Many human functions show a marked decline prior to death during a period ranging from a few weeks to a few years. [8,9] Gerontologists have called this phenomenon terminal drop, a determinant chain of functional changes that are due to a death process. Terminal drop in physical and cognitive function have been studied extensively. One such study documented a significant terminal drop in the last 5 years of life for depression and self-rated health in persons over 65. [10] In the last year of life, men fared better than women in each age group, but the trends with age were unclear. We have not located any studies of terminal drop in quality of life, but as QOL has been found to be highly correlated with both depression and self-rated health, [11] it seems likely that QOL at the end of life will also exhibit a terminal drop and be better for men. The expected relationship with age is unclear.

## 2.0 Methods

Data came from the ongoing Complementary Comfort Care randomized trial of complementary and alternative medicine at the end of life, sponsored by the National Cancer Institute (William Lafferty, PI). Enrollees are randomized to receive massage, guided meditation, or a friendly visit. For this paper, we ignored treatment group assignment and considered all persons enrolled to date as a single group.

### 2.1 Study Participants

Study participants were recruited from four Seattle-area hospice organizations and other medical care sites that were likely to see persons with advanced medical conditions who were near the end of life, as well as through support groups and personal networks. Persons who were 18 years of age or older, English speaking, able to report reasonably accurately on symptoms and quality of life for the previous 7 days, willing to accept assignment to any of the three treatment arms, and who had a family member or friend who would act as their “study partner” to provide ancillary data to the study were eligible to participate, regardless of specific diagnosis. Enrollees and their study partners were paid $25 after completing baseline interviews. Longitudinal information on quality of life and symptom status was collected either in person or by telephone after every two intervention visits. The goal was to provide up to two intervention visits per week, and persons were allowed to reschedule or to drop out of the study at any time.

### 2.2 Outcome Measures

Although there are many instruments for measuring QOL, this study needed to minimize subject burden. We collected only 6 items from the Perceived Quality of Life survey, [2] and for this paper report on only a single item. Patients were asked “How would you rate your over-all quality of life during the last 7 days?” from 0 for no quality of life to 10 for perfect quality of life. Here, QOL is defined as the person's rating, which was collected after every 2 intervention visits (approximately every week). Participants also rated their health status, at baseline only, on the same 0–10 scale. A 6-point rating of the presence and severity of pain was also obtained at each interview. There is no recommended way to code “dead” on any of these measures, which is a potential problem in studies where some persons die.

To estimate the reliability of the QOL measure in a stable population, we compared QOL values made one week apart for persons whose pain rating did not change in that week. The intraclass correlation coefficient (test-retest reliability) was 0.73, which is acceptable for our purposes. We arbitrarily defined a value of 7–10 as “good QOL”. The intervention providers were also asked to estimate the prognosis in terms of QOL for about 30 persons who stopped providing data substantially before death or the analysis date, to help inform our later sensitivity analysis of the imputation model.

### 2.3 Study sample for the current paper

This paper deals with the subset of data available in February, 2007 (referred to as the analysis date). We eliminated 15 persons whose vital status was not then known (not known to be dead, but had provided no data in the most recent 45 days). The prospective sample included all persons who enrolled 12 or more months before the analysis date. The retrospective sample included all persons who died 10 weeks or more after enrollment. Persons could be in both samples.

### 2.4 Accounting for Death and Missing Data

Longitudinal data at the end of life are often difficult to collect and interpret because of varying lengths of follow-up, different dying trajectories, and missing observations. Some persons had no QOL data at, say, 52 weeks, because they were recruited less than 1 year before the analysis date, had died, were not scheduled to provide data that week, or had missing data for some other reason. The average of all available QOL value at 52 weeks is thus meaningless. We needed to account for death and for other missing data.

One way to account for death is to transform the original variable that has no value for death into a new variable that does have such a value. [13-16] Here we transformed QOL to the probability that a person will have good QOL next week (have QOL ≥ 7 next week), estimated from his QOL this week. We used all transition pairs (two values of QOL for the same person 1 week apart) in the longitudinal data, and estimated the transformation parameters from a logistic regression (using data from the first 148 enrollees) of a binary variable “Good QOL 1 week later” on “QOL now”. The regression results were as follows:

logit (Good QOL 1 week later) = -4.180 + .680*QOL now.

The estimated probability of having good QOL 1 week later as a function of current QOL is then:

QOLt=exp⁡(−4.180+0.680*QOL)1+exp⁡(−4.180+0.680*QOL),
 MathType@MTEF@5@5@+=feaafiart1ev1aaatCvAUfKttLearuWrP9MDH5MBPbIqV92AaeXatLxBI9gBaebbnrfifHhDYfgasaacH8akY=wiFfYdH8Gipec8Eeeu0xXdbba9frFj0=OqFfea0dXdd9vqai=hGuQ8kuc9pgc9s8qqaq=dirpe0xb9q8qiLsFr0=vr0=vr0dc8meaabaqaciaacaGaaeqabaqabeGadaaakeaacqWGrbqucqWGpbWtcqWGmbatcqWG0baDcqGH9aqpdaWcaaqaaiGbcwgaLjabcIha4jabcchaWjabcIcaOiabgkHiTiabisda0iabc6caUiabigdaXiabiIda4iabicdaWiabgUcaRiabicdaWiabc6caUiabiAda2iabiIda4iabicdaWiabcQcaQiabdgfarjabd+eapjabdYeamjabcMcaPaqaaiabigdaXiabgUcaRiGbcwgaLjabcIha4jabcchaWjabcIcaOiabgkHiTiabisda0iabc6caUiabigdaXiabiIda4iabicdaWiabgUcaRiabicdaWiabc6caUiabiAda2iabiIda4iabicdaWiabcQcaQiabdgfarjabd+eapjabdYeamjabcMcaPaaacqGGSaalaaa@603C@

(where QOLt refers to QOL-transformed). For example, a QOL of 10 corresponded to a QOLt of .932, and a QOL of 0 corresponds to a QOLt of 0.015. The probability that a dead person will have good QOL a week later is clearly 0, which provides a value after death. We set the values of QOLt for the weeks after a person died to zero, with the new variable referred to as QOLtd (QOL transformed with deaths added).

QOL for weeks when persons were alive but had no QOL data were imputed from the regression of QOLtd on the logarithm of time from death (or time from the analysis date if still alive), as explained in detail in Appendix 1 and considered further in the discussion section. An example of the missing data is given in the following section.

### 2.5 Data Organization

The plan was for persons to receive up to two treatment interventions per week, but some persons preferred to receive one intervention per week, and so were scheduled to provide QOL data every two weeks. Some persons skipped weeks and/or dropped out of the intervention. As a result, each person had a unique data collection schedule. The steps required in preparing this complex dataset for analysis (transform, account for death, impute missing data – “tdi”) are explained by example here, and in more detail in Appendix 1. An assessment of the sensitivity of results to imputation of missing data, is described below in section 3.4 and in more detail in Appendix 2.

**Figure 1:**
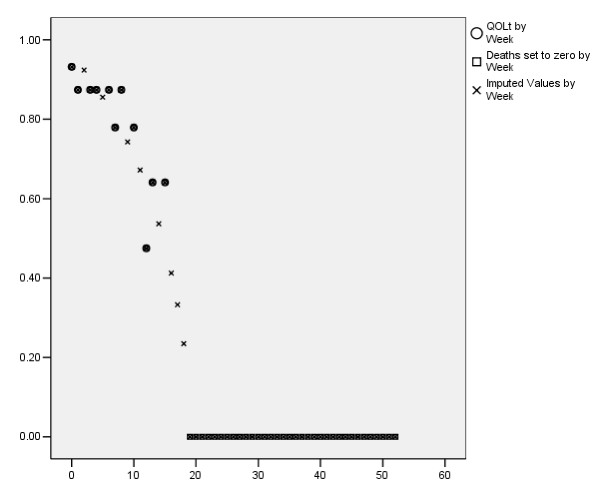
Quality of Life over time for "Mr. Smith".

The data for one participant, “Mr. Smith”, are shown in Table 1. This gentleman was enrolled in the study 137 weeks before the analysis date, but survived for only 18 weeks. He provided eleven QOL assessments between week 0 (his enrollment week) and week 15 (shown in column 3).QOLt is the transformed QOL value (the estimated probability of having good QOL in the following week conditional on his current QOL), which is in column 4. [16] As noted above, a person with a QOL of 10 has probability 0.93 of having good quality of life one week later (or, put another way, about 93% of persons with a QOL of 10 had QOL ≥ 7 one week later). The probability that a dead person will have good QOL a week later is zero, and so column 5 includes a zero for each week after he died (QOLtd). In column 6 the remaining missing values were imputed from a regression of Mr. Smith's data on the log of time from death, as explained in Appendix 1 (QOLtdi). Note that Mr. Smith had no real QOL data in the three weeks before death, and is set to missing from weeks 138 to 150 because his potential follow-up was only 137 weeks. Figure 1 shows Mr. Smith's data in the first 52 weeks; a circle is observed data, an x represents imputed data, and a square represents the zeroes after death.

**Table 1:** Longitudinal Data for “Mr. Smith” (WQL = 12.63 prospective, = 5.10 retrospective)

1	2	3	4	5	6	7
**Weeks after Baseline**	**Weeks Before Death**	**QOL**	**QOLt**	**QOLtd**	**QOLtdi**	**QOLback**

0	-18	10	.93	.93	.93	10.00
1	-17	9	.87	.87	.87	9.00
2	-16	.			.92	9.82
3	-15	9	.87	.87	.87	9.00
4	-14	9	.87	.87	.87	9.00
5	-13	.			.86	8.77
6	-12	9	.87	.87	.87	9.00
7	-11	8	.78	.78	.78	8.00
8	-10	9	.87	.87	.87	9.00
9	-9	.			.74	7.71
10	-8	8	.78	.78	.78	8.00
11	-7	.			.67	7.20
12	-6	6	.48	.48	.48	6.00
13	-5	7	.64	.64	.64	7.00
14	-4	.			.54	6.36
15	-3	7	.64	.64	.64	7.00
16	-2	.			.41	5.63
17	-1	.			.33	5.13
18	0	.			.23	4.41
19	.	.	.	0	0	d
20	.	.	.	0	0	d
21	.	.	.	0	0	d
.		.	.	0	0	d
.		.	.	0	0	d
137	.	.	.	0	0	d
138	.	.	.	.	.	.
.	.	.	.	.	.	.
150	.	.	.	.	.	.

1 Weeks after enrollment (0 is person's baseline)

2 Weeks before death. (0 is the week the person died)

3 QOL: Observed QOL data

4 QOLt: QOL transformed to the probability of having good QOL next week given current QOL

5 QOLtd: QOLt with the weeks when person was dead set to 0. Weeks 138–150 are missing because Mr. Smith enrolled only 137 weeks before the end of current data collection.

6 QOLtdi: QOLtd with missing data imputed. Unknown for weeks 117–131 (for Mr. Smith).

7 QOLtdi transformed back to the original categories. (identical to observed QOL values, imputed values may need to be rounded).

For column 7, the QOLtdi data were transformed back to the original scale, and the values of “QOLback” are shown. There are more values in column 7 than in column 3, and not all of them are integers, because of the re-transformation of the imputed QOLtdi data. There are no numerical values after death, but “d” flags are included to indicate weeks when he was dead. More detail is given in Appendix 1.

If we plotted the QOLtdi values in column 6 from week 0 to week 52 against time, as in Figure 1, the area under this curve would be the estimated number of weeks of good-quality life in the year after enrollment. This area was calculated, using the trapezoidal method, as the sum of those 53 QOLtdi values, minus half of the first and last values. [17] Mr. Smith had the equivalent of 12.63 weeks of good-quality life (WQL). Weeks of good-quality life in the 10 weeks before death are the sum of QOLtdi from week -9 to the first zero QOLtdi minus half of the first and last values. Mr. Smith experienced the equivalent of 5.10 weeks of good-quality life in the 10 weeks before his death.

### 2.6 Analysis

We used several graphical methods to describe QOL in the 52 weeks after enrollment and in the 10 weeks before death. We estimated the area under the curve of QOLtdi over time, which is interpreted as (expected) weeks of good-quality life, or WQL. (WQL is conceptually similar to quality-adjusted life years (QALY) except that it is measured in weeks, and is based on QOLtdi, which is not a preference-rated measure). We regressed WQL on age, log age, sex, age*sex, log age * sex, education, cancer, and the baseline values of QOL, Pain, and Health status, using backward elimination to obtain a parsimonious model. For the retrospective regression analysis we also added length of survival as a potential covariate, because the baseline values were likely to be less salient for a person with lengthy survival. Interactions between survival and baseline values were also included.

## 3.0 Findings

### 3.1 Sample description

At the analysis date, 167 persons had entered the trial. Median survival was 149 days, 165 for persons under age 60 at baseline and 130 for those 60 and older. The following analysis will deal with the 115 persons who had been enrolled at least 1 year as of the analysis date, and the 83 who died after being enrolled at least 10 weeks. Of these, 72 persons were eligible for both analyses.

### 3.2 Prospective analysis: QOL in the year after randomization (N = 115)

#### 3.2.0 Description of Prospective Sample

Column 1 of Table 2 contains descriptive statistics for the prospective sample. Age ranged from 36 to 98 (mean = 70, s.d. = 16) and 70% were age 60 or older. Ten were still alive 1 year later, and their mean age was 61 as compared to 71 for those who had died. Most of the subjects (63%) were women. Mean QOLtdi changed from .53 at baseline to .10 one year later, representing a drop of .43 in the estimated proportion with good QOL.

**Table 2:** Baseline Characteristics of Prospective and Retrospective Samples

		**Prospective**	**Retrospective**
**N**		115	83
			
**Age**	Mean	69.78	69.39
	< 60 (#)	35	26
	≥ 60	80	57
			
**Gender**	Male (#)	43	31
	Female	72	52
			
**Race**	Non-white (#)	10	8
	White	105	75
			
**Education**	< College (#)	64	47
	College graduate	51	36
			
**Monthly Income**	< $2000 (#)	39	24
	≥ $2000	62	51
	Missing	14	8
			
**Referral source**	Other source (#)	24	20
	Hospice	91	63
			
**Cancer**	other dx (#)	37	25
	cancer	78	58
			
**Baseline Health (0–10, high best)**	Mean	5.72	5.53
			
**PAIN Baseline (0–6, low best)**	Mean	2.35	2.29
			
**QOL Baseline (0–10, high best)**	Mean	6.23	6.16
	Proportion ≥ 7	.56	.55
			
**Vital Status at analysis date**	Dead (#)	105	83
	censored	10	0

#### 3.2.1 QOLback in the Prospective Sample

**Figure 2:**
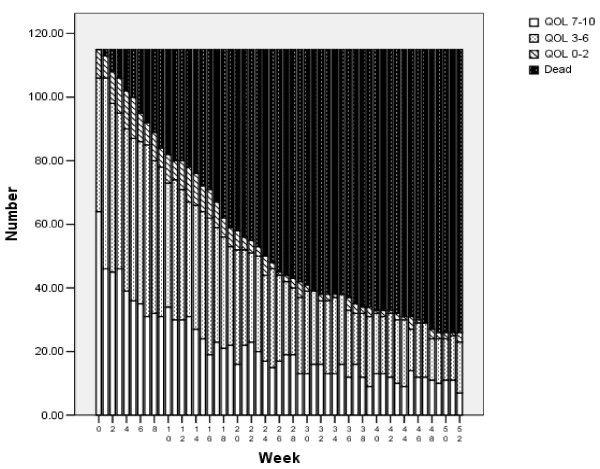
Quality of Life in 12 months after baseline (back-transformed, N = 115).

We first examined QOLback, the re-transformed QOLtdi data; that is, QOLtdi was restored to the original QOL scale (which has no value for death). Mean QOLback has no longitudinal interpretation because it refers to different people each week (those still alive at that time). In Figure 2 we resolved this by maintaining a separate category for dead. All 115 persons have information at each week (whether real, imputed, or dead), and so longitudinal conclusions are appropriate. The lowest (lightest) bars represent the number with good QOL (7–10), the other shadings represent moderate QOL (3–6) and low QOL (0–2), and the highest (darkest) bars are the number who were dead by that week. More than half reported good QOL at baseline. The number dead of course increased over time, while the others decreased. Perhaps surprisingly, there were always persons with good or moderate QOL, and the number with the worst QOL was small at all times. These population trajectories over time are fairly smooth and might be easy to model.

#### 3.2.2 QOLtdi in Prospective Sample

**Figure 3:**
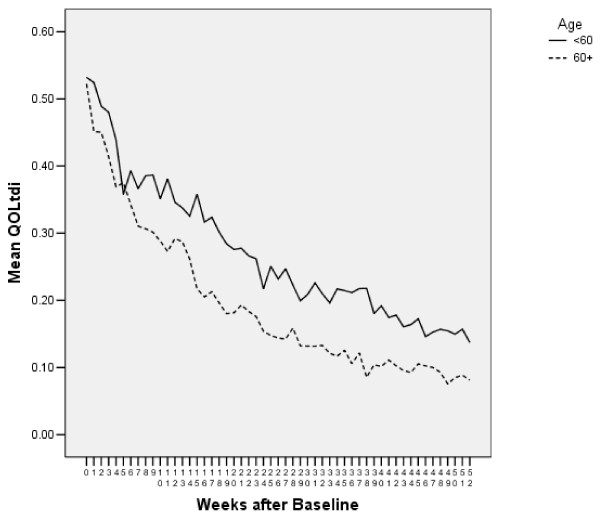
Mean QOLtdi in 12 months after baseline, by age (N = 115). The dotted line is age ≥ 60, the solid is age < 60.

Although Figure 2 gives an excellent picture of the distribution of QOL over time, such graphs do not provide a convenient way to investigate the relationship of QOL to other variables, such as age. Figure 3 shows the mean QOLtdi each week for persons below and at or above age 60. Mean QOLtdi is the estimated proportion of the cohort who had good QOL each week. (Technically, it is an estimate of the proportion expected to have good QOL in the following week, but we ignore this detail for simplicity). The area under each curve is the average number of weeks of good-quality life (WQL). Overall, mean WQL was 11.1 weeks, median WQL was 6.8 weeks, and 13% of the enrollees had less than one cumulative WQL in the year following enrollment. Mean WQL was 13.8 weeks for persons under 60, and 9.8 for persons 60 and above. The lines for the two age groups were similar at baseline but diverged after a few weeks. (This pattern occurred for the QOLt data, as well, suggesting that the age finding is not an artifact of our handling of death and missing data).

#### 3.2.3 Regression results in the Prospective Sample)

The first 2 columns in Table 3 describe the correlation and regression of WQL on the listed covariates. We used all of the variables in Table 2 but income (because of missing data), and referral source (because the great majority were in hospice at baseline or shortly thereafter). Column 1 shows the correlation of each baseline variable with WQL, with superscripts indicating that WQL was significantly higher for persons with better baseline QOL. The regression coefficients from the backwards elimination regression of WQL on the covariates are shown in column 2. The procedure retained baseline QOL and the dummy variable representing age ≥ 60. From the regression equation, WQL increased by 1.7 weeks for each additional point of baseline QOL, and persons aged 60 or over had 3.8 fewer WQL in the 52 weeks after enrollment.

**Table 3:** Correlation^1 ^and Regression^2 ^Results

	**Prospective**		**Retrospective**	
	**1**	**2**	**3**	**4**

	**Correlation**	**Regression**	**Correlation**	**Regression**

**R**^2^		.17		.37
**Age**	-0.13		0.09	
**Age ≥ 60**	-0.17	-3.81	0.16	0.75
**Gender**	0.05		0.07	
**White**	-0.04		-0.00	
**College Graduate**	-0.01		-0.18	
**Log (age)**	-0.14		0.08	
Age*sex	0.03		0.11	
Log (age)*sex	0.05		0.08	
**Cancer**	-0.03		0.03	
**Baseline health (high best)**	0.17		0.25^3^	
**QOL Baseline (high best)**	0.38^3^	1.70	0.58^3^	0.43
**PAIN Baseline (low best)**	-0.01		-0.04	
**# of days lived**			0.08	
Days Lived*QOL			0.32^3^	
Days Lived*Pain			.14	

1 Correlation with WQL.

2 Regression of WQL on the column variables, using backward elimination with a p to retain of 0.10.

3 Correlation coefficient is significantly different from zero (p < .05 2-tailed).

### 3.3 Retrospective analysis: QOL in 10 weeks before Death (N = 63)

#### 3.3.0 Description of the Retrospective Sample

Descriptive statistics for the retrospective sample are in column 2 of Table 2. This analysis included only the 83 persons who died after having survived at least 10 weeks. Age ranged from 36 to 98 years, and mean and median age were 69 and 71, respectively. Women comprised 63% of this sample.

#### 3.3.1 QOLback in the Retrospective Sample

**Figure 4:**
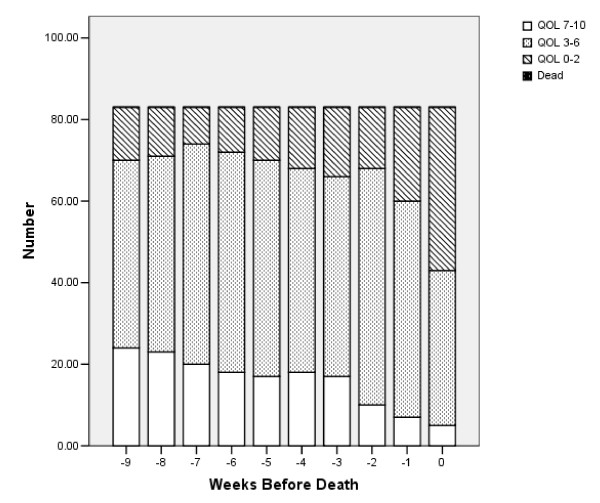
Quality of Life in 10 weeks before death (back-transformed, N = 83).

Figure 4 is comparable to Figure 2. It shows the number of persons in each QOL category (re-transformed from QOLtdi) within 7 days of death (week 0) as well as in the 9 weeks prior. Nearly 30% had good QOL (7 to10) 10 weeks before death, but that the number dropped sharply in the 3 weeks before death. Nearly half had poor QOL in the week just before death.

#### 3.3.2 QOLtdi in the Retrospective Sample

**Figure 5:**
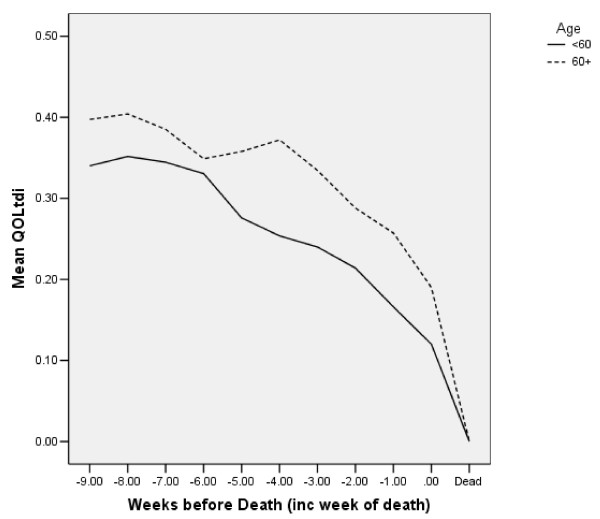
Mean QOLtdi in 10 weeks before death, by age (N = 83). The dotted line is age ≥ 60, the solid is age < 60.

Figure 5 shows the average QOLtdi by age group in the week of death and in the ten preceding weeks. The area under the QOLtdi curve is the number of weeks of good-quality life in the 10 weeks before death. Mean WQL was 2.92 over all, and was 2.45 for age <60 and 3.14 for age ≥ 60. Thus, the older group had better WQL in the retrospective sample. (This pattern also occurred for the QOLt data, suggesting that the age finding is not an artifact of our handling death and missing data).

#### 3.3.3 Regression Analysis (retrospective sample)

Regression and correlation results for the retrospective analysis are in columns 3–4 of Table 3. Bivariate correlations show that WQL (weeks of good-quality life) was significantly associated in the expected way with baseline health status and QOL. Older persons had higher WQL, but not significantly so. The significant interaction indicated that persons who had higher QOL at baseline and also lived longer had more WQL near death.

A backward elimination regression of WQL on the regression variables retained baseline QOL and the dummy variable for age ≥ 60. WQL increased 0.43 weeks for each additional point of baseline QOL. The older persons had 0.75 more adjusted WQL than younger persons in the 10 weeks before death, consistent with Figure 5. At baseline, QOL was higher for the younger group (6.3 vs 6.1 for younger and older, respectively). Mean WQL was 2.45 weeks for persons under 60 and 3.14 for persons ≥ 60. Among the 72 persons eligible for both studies, the younger had slightly lower WQL in the retrospective analysis (2.51 vs. 3.12) and slightly higher WQL in the prospective analysis (13.96 vs. 12.69). Some persons in the prospective analysis were still alive on the analysis date, and thus not eligible for the retrospective analysis. It may not be surprising that results for age are different for the two analyses. A sensitivity analysis is discussed next.

### 3.4 Sensitivity analysis for missing data imputation

There was a good deal of “missing” data, due in part to persons not being scheduled in a particular week, or possibly for reason related to the person's QOL. We felt that the most serious threat to validity was data imputed incorrectly between the last observed value and death. For example, 19% of the persons in the retrospective study had no real data in the last 10 weeks of life, and another 25% had only 1 to 3 real values. Persons under 60 averaged only 2.8 real values in their last 10 weeks of life, while those 60 and older averaged 5.0 real values. For these reasons, the possible effect of missing data on the previous findings was explored.

As a sensitivity analysis we imputed missing data in four different ways: regression of QOLtd on log of time from death (QOLtdi), carrying the last known observation forward in time until death or the analysis date (LOCF), setting all values after the last known value to half of the minimum QOLt ever observed (Half Min), and simple linear interpolation (for persons who died only). As detailed in Appendix 2, the results of the prospective study were very similar whether we used QOLtdi, LOCF, or Half Min. For the retrospective study, findings for QOLtdi and linear interpolation were very similar, but for Half Min there was no age effect, and for LOCF there was no age effect or terminal drop. The prospective study findings are thus robust to the imputation method, but the retrospective study findings are more sensitive.

## 4.0 Summary/discussion

We have observed some features of quality of life at the end of life, and also illustrated the complexities of this type of longitudinal data. We first discuss the substantive findings, and then comment on the methodology. Readers interested mainly in the substantive results may prefer to omit section 4.2.

### 4.1 QOL at the End of Life

There were several interesting findings in this exploratory study. Median survival was nearly 5 months, although hospice statistics had suggested it would be 3 months. This suggests that there was a “healthy volunteer” bias even at the end of life. Throughout the first 52 weeks, there were always a substantial number of persons whose quality of life was good (7–10), and never an excess of persons with the worst quality of life. Persons in the lowest QOL category had a higher mortality rate, and thus did not linger in that state. There may also have been a response shift, in which persons began to rate their QOL differently over time. [18]

Figures 4 and 5 show a sharp decrease in QOL in the three weeks just before death. This is referred to as “terminal drop”, and has been noted in many other settings. If this three-week period could be identified in advance, it might be possible to develop interventions for that period. However, it is difficult to predict in advance the very end of life.

The area under the curves is interpreted as WQL = weeks of good-quality life. Baseline health status, QOL, and pain were all associated in the expected way with WQL. In this small sample, the expected association of WQL with gender was not found, nor was there an association with race or with having cancer. This suggests that the future QOL of dying persons may be independent of many of the patient characteristics that are associated with future QOL in healthier populations.

The association of age with QOL was also interesting. Younger persons had higher QOL at baseline than older persons, and in the longitudinal analysis WQL was higher for younger persons, but in the retrospective analysis, younger persons had lower WQL. There are several possible technical explanations for these findings. Because younger persons lived longer, they also had more time to accumulate WQL than the older persons in the prospective study, while everyone was counted for exactly 10 weeks in the retrospective study. The prospective analysis also included persons who had not yet died, which could have caused differences. Finally, the age reversal could be an artifact of the missing data imputation, because the younger persons had fewer real observations in the last 10 weeks of life than the older persons. In the sensitivity analysis for the retrospective sample, the age effect held for the two imputation methods that assumed a gradual decline from the last observed value until death, but was not observed for the method that assumed a sharp drop at the time at of the last observed value (Half Min), or a sharp drop at the time of death (LOCF). If the age relationship found here does hold up in other studies, it could suggest that special interventions are needed to support the QOL of persons approaching a premature death (operationally defined here as death before age 60). This finding is consistent with the ambiguous results about age reported elsewhere for depression and self-rated health in the last year of life. [10]

## 4.2 Methods Results

### Transformation

In this complex dataset, we used the approach of “transform to a scale with a value for death, add values for death, impute missing data” (“tdi” or “tidy”), to obtain a tidy dataset that accounts for the status of every person for every week after enrollment. The figures and regressions used various versions of the data (columns 6–8 in Table 1). Deaths were either included (QOLtdi), accounted for as a separate category (QOLback) or were not applicable (in the retrospective analysis). The tidy dataset supports all of these approaches.

Other ways to transform the data have been suggested. [15] The simplest method is to transform the QOL data to a binomial variable that takes the value 1 for QOL = 7 through 10 and 0 for lower values (or death). This is easy to interpret (good QOL yes/no) and gives the actual years of good-quality life, equivalent to the sum of the lowest bars in Figure 2. Because it combines the QOL categories, however, it can not be back-transformed to the original scale. Making no distinction between being dead and having a QOL < 7 is also unsatisfying. For example, a person with a value of 7 every week would have 52 weeks of healthy life, while a person with 6 every week would have zero weeks under this transformation. (Under the QOLtdi transformation used in this paper, the person with 7's every week would have 33 WQL and the person with 6's would have 25 WQL). Further, any intervention that made improvement in the lower part of the QOL scale, such as moving persons from a QOL of 2 to a QOL of 6, would receive no credit under this scheme. Using the binary transformation, mean WQL was 8.0 (vs. 11.1 using QOLtdi) in the prospective study and 1.6 (vs. 2.9) in the retrospective study. Mean WQL was lower because there was no “partial credit” for QOL values under 7. The prospective regression using the binary transformation retained the same two variables as in Table 3, The retrospective regression retained only baseline QOL, although age ≥ 60 was the last variable to be eliminated. Thus the main results would have been similar if we had used the binary transformation, but the age effect is less clear in the retrospective study.

We also considered a third approach that transformed QOL to the probability of having a health rating ≥7 this week (rather than QOL ≥ 7 next week). This transformation also gave essentially the same findings as reported in the main analysis, although weeks of “healthy” life were lower than “weeks of good-quality life”.

### Imputation

There are many ways to impute missing data, and no agreement about the best method for longitudinal data. As detailed in Appendix 1, we assumed that the missing values fell on the line of the regression of QOLtdi on log(time from death), with a different intercept and slope for each person. This imputation model was supported in the observed data, but is of course only an approximation. Other approaches that used each person's observed longitudinal data and the date of death to impute missing data gave similar results. Some analytic approaches such as GEE longitudinal analysis of QOLtd would not need the imputed data because such analyses implicitly impute the missing data. (But analysis of QOL or QOLt rather than QOLtd would have the unfortunate effect of implicitly imputing QOL after the person died). Multiple imputation was not used here because our summary measure was the area under the curve, which was much the same with and without the imputed data. Sensitivity analysis showed that the imputation method was not important for the prospective analysis but that the retrospective findings were less robust.

### Modeling QOL over time

The trends over time shown in the Figures suggest that population trends were non-linear but fairly smooth (see Figure 2 and Figure 3). The person-level QOLtdi curve would be more difficult to model because it starts with a backward S shape which becomes flat after death (See Figure 1). Our dependent variable, WQL, did not require fitting the trends over time at either the person level or the population level, but only an estimate of the area under the curve for each person.

## 4.3 Limitations

The prospective analysis used only the persons who enrolled early in the main study, and the retrospective analysis was restricted to those who lived at least 10 weeks. The results may not generalize to all patients at the end of life or even to all persons enrolled in the main study. The measure of QOL used here is not the only or even the most popular measure of QOL, and other instruments may find different trends over time. Imputation methods for the missing data were of some concern for the retrospective analysis. The sample sizes (115 prospective and 83 retrospective) are not large. The results shown here are primarily descriptive, and should be considered as hypothesis generating rather than definitive.

## 4.4 Conclusion

This exploratory analysis is the first to describe the trajectory of quality of life at the end of life in such detail. Although we found a terminal drop in QOL in the final 3 weeks of life, and particularly low QOL for persons approaching a premature death, other studies are needed to explore these findings further. The methodology described here may be useful to other researchers as well.

## Appendix 1

### Data Collection and Organization

To organize these longitudinal data we created a vector (column) for each participant with one space for every week of that person's potential follow-up (that is, for every week between their enrollment and the analysis date – July 1, 2007). This appendix corresponds with Table 1, which shows the information for Mr. Smith.

#### Column 1: Week

Weeks after enrollment, where day 0 is enrollment day, and days 0–6 are week 0.

#### Column 2: Weeks before death

Week before death = week minus the integer part of (# days survived/7) + 1.

#### Column 3: QOL

QOL is self-rated quality of life on a scale from 0 (no QOL) to 10 (perfect QOL). QOL in week 0 is the baseline QOL. On the rare occasion when a person has two interviews in a week, the mean of the two values is used (resulting in some non-integers).

#### Column 4: QOLt

QOL does not have a value for death. To incorporate death into this measure, we transformed QOL into QOLt, which is the (estimated) probability that the person will have good QOL (QOL ≥ 7) one week later, based on his current QOL value [16]. The choice of 7, though arbitrary, has face validity and a reasonable number of persons were above and below 7 at baseline. The transformation was derived by a logistic regression of “Good QOL 1 week later” on QOL now. The regression results were as follows: logit (Good QOL 1 week later) = -4.180 + .680*QOL now, or a+b*QOL for short. The estimated probability of having good QOL next week as a function of current QOL is Prob(Good QOL 1 week later | QOL) = exp(a+b*QOL)/(1 + exp(a+b*QOL)). For example, a QOL of 10 corresponded to a QOLt of .93, and a QOL of 0 corresponds to a QOLt of .015. The interpretation of QOLt is the estimated probability that this person will have good QOL in the following week. The interpretation of the average QOLt for some group is the estimated proportion of people in that group who will have good QOL a week later. The circles in Figure 1 are the QOLt data for Mr. Smith, plotted against time.

#### Column 5: QOLtd

For weeks after death, persons received a zero because they had no probability of having good QOL one week later (QOLtd). The squares in Figure 1 represent the dead weeks, out to the end of Mr. Smith's potential follow-up, which was 137 weeks (only the first 52 weeks are shown).

#### Column 6: QOLtdi

QOLtdi is QOLtd, but uses imputed values for weeks when the person was alive but did not provide data. Although there is some agreement about imputation methods for cross-sectional data, there is no standard approach for longitudinal data. We consider the missing data mechanism to be missing at random for some observations (e.g., those missing because the person initially chose an every-other-week schedule) and missing not-at-random for other observations, most importantly those that were missing between the last real observation and death.

Engels and Diehr examined the performance of 14 simple imputation methods in a dataset of older adults. They found that all 14 methods provided estimates that were too optimistic, and that estimates based on the person's observed longitudinal data had lower mean squared error than other types of estimates. We chose an imputation strategy that would reproduce, as well as possible, the QOLtd data from baseline up to the first week after death (the first zero). Since the first and last values are known for each person who dies, this may be considered closer to interpolation than imputation.

Data were imputed by regressing observed QOLtd on the logarithm of the number of weeks before this person died. Log(weeks from death) was chosen based on examination of scatterplots for the first 20 persons who died and had at least 10 real values, for different transformations of time. Weeks with missing data were set to the regression estimate for that time. Only the first “zero” was used in the regression. That is, for Mr. Smith, only the QOLtd values in weeks 0 to 19 were used in the imputation regression.

**Figure 6:**
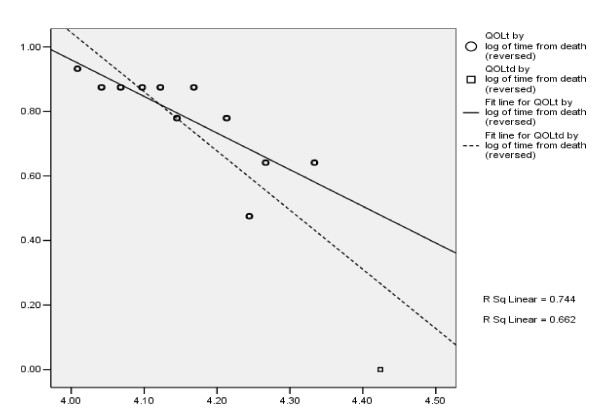
Imputation regression for "Mr. Smith".

Figure 6 shows the imputation regression line used for Mr. Smith. All of the QOLtd values but the “zero” value were calculated from observed QOL data. (The X axis is reversed so the regression line in this Figure will decrease over time, similar to the other Figures). The solid regression line uses only the observed data. The dashed regression line uses the observed data plus the first zero for death, and is the line used to impute missing data for Mr. Smith. Each person had a different imputation regression. In regressions of QOLtd over time, the zero representing the week of death (the first week in which data were not received because the person died) is an influential point, which should tend to counter the positive bias noted above. Using a person's available longitudinal data to estimate the missing data worked well for older persons in one setting, suggesting that it may be appropriate for dying people as well. Mr. Smith had an imputation slope of 32.7 and an imputation intercept of -91.8. In Figure 1 the x's represent the imputed values.

By this scheme, the imputed values are monotonically decreasing in time for persons who die, but within the range of the observed values persons may improve (an imputed value may be higher than the previous observed value). Persons who have not yet died may have stable or improving imputed values, depending on the trend of their observed data.

We considered multiple imputation (such as adding random values drawn from the distribution of residuals to the regression estimate to create several imputed values for each missing observation). However, we felt that this would not deal with the riskiest part of the imputation, the period between the last real value and death. This period was only 3 weeks long for Mr. Smith but was longer for many other persons. We instead conducted a sensitivity analysis by creating other versions of QOLtdi, as explained in Appendix 2.

#### Column 7: QOLback

QOLback = QOLtdi re-transformed to the original scale, by inverting the logistic regression equation.

QOLback = (logit(QOLt) - a)/b = [ln ((QOLt)/(1-QOLt)) - (-4.180)]/.682.

### Weeks of Good-quality life (Area Under the Curve)

The area under the curve in Figure 1 is the number of weeks of good-quality life (WQL) (estimated weeks spent with QOL ≥ 7). For the 52-week WQL discussed in the paper, WQL is the sum of the QOLtdi values from week 1 to week 51, plus half the value at week 0 plus half the value at week 52. [17] The “half” multipliers are a feature of the trapezoidal method, which uses every value twice except the first and the last.

## Appendix 2

### Sensitivity analysis for missing data imputation

There was a good deal of “missing” data, due to persons not being scheduled in a particular week, or for other possibly QOL-related reasons. We felt that the most serious problem was data imputed between the last observed data and death. Of the 83 persons in the retrospective study, 16 (19%) had no real data in the 10 weeks before death, and 25% had only 1 to 3 real values. Persons under age 60 averaged 2.8 real values in their last 10 weeks of life, while those over 60 averaged 5 real values. If a person has several real values in the last ten weeks of life, the imputed values will be consistent with those observed values. However, with only 0 or 1 real values, the imputed data will depend strongly on trends in the earlier part of the data and on the imputation model.

For these reasons, we considered four different ways of imputing missing data between the last real value and death. The four methods were: (i) using the regression on log of time from death (or the analysis date); (j) carrying the last known value forward in time until death (or the analysis date); (k) setting all values after the last known value to half of the minimum QOLt ever observed; and (l) a simple linear interpolation of all the QOLtd values (not just those after the last real value, and for persons who died only), on the natural time scale. For example, in Table 1, Mr. Smith's last real QOLtdi value was .64, followed by the imputed values .41, .33, and .23 (imputation method i). Under the other imputation methods those 4 values are: (j) .64 .64 .64 .64; (k) .64 .24 .24 .24; and (l) .64 .48 .32 .16. His four related values of WQL in the prospective study were 12.63, 13.57, 12.36, and 12.37; in the retrospective study, his four values were 5.10, 6.04, 4.83, and 5.18.

For the prospective analysis, we compared methods i, j, and k. (Not every still-living person had a “final” value, and so linear interpolation was not possible). The average WQL values for these different datasets were 11.1, 11.6, and 11.2 respectively. Not surprisingly, imputation j resulted in more good quality weeks, and imputation k had fewer, than imputation i. Figures 2 and 3 were similar under all 3 imputation schemes. The backwards selection regression chose the same variables for all methods of imputation. The method of imputation influences the means, but does not seem to be a concern for the relationships in the prospective analyses.

For the retrospective analysis we compared all four imputation methods. The average WQL values were 2.9, 3.6, 3.0, and 2.7 for methods i, j, k, and l, respectively. WQLj was higher than the others, because it assumed no decrease in QOL from the last observed measure to the time of death. It will be discussed separately. Figures 3 and 4 were very similar for imputations i and l, and showed no age effect for method k. Regressions for i and l retained baseline QOL and age > 60. Method k retained only baseline QOL.

Method j (last real observation carried forward until death) was quite different. In the equivalent of Figure 5, the younger had better QOL than the older. There was no terminal drop and older persons had lower QOL than the younger persons. The only variable to remain in the regression was baseline QOL. The retrospective results were thus more sensitive to the type of imputation than were the results of the prospective analysis. Most persons who stopped providing data did so because they were too sick to participate further, but there were a few persons whose health did improve. The intervention providers were asked to estimate whether the QOL trajectory for persons who provided no data near the end would be a little better, the same, worse, or much worse. They identified only 1 person who would probably be better and another would stay the same. We think it likely that QOL usually declined after a person stopped providing data. Two of the four imputation methods supported the age effect in the retrospective analysis, as did 3 of 3 for the prospective analysis. However, all of the findings need replication.
